# Selective Toxicity of Iron, Manganese Oxide Nanostructure and Laser Wave on Colorectal and Breast Cancer Cell

**DOI:** 10.5812/ijpr-157301

**Published:** 2025-04-29

**Authors:** Aysan Mansournia, Nasim Nobari, Fatemeh Ghanbary, Behrooz Khezri

**Affiliations:** 1 Department of Physic, Mahabad Branch, Islamic Azad University, Mahabad, Iran; 2 Department of Chemistry, Mahabad Branch, Islamic Azad University, Mahabad, Iran

**Keywords:** Cell Death Signaling, Apoptosis, Nanostructure, Stress Oxidative

## Abstract

**Background:**

Cancer is a deadly and multifaceted disease that poses a significant challenge to treatment due to its heterogeneity and ability to adapt and evolve. Despite advancements in research and medicine, the development of effective treatment options remains a major obstacle in the battle against cancer. Manganese oxide (MnO) and iron (III) oxide (Fe_2_O_3_) nanoparticles (NPs) are increasingly used for numerous new applications in modern industrial sectors. However, the toxic and treatment impact of MnO and Fe_2_O_3_ NPs has not been clearly elucidated on human cell lines at the cellular and molecular levels.

**Objectives:**

This study aimed to assess the potential cytotoxic effect of combining infrared (IR) laser therapy with MnO and Fe_2_O_3_ nanoparticles on breast and colorectal cancer cells for cancer treatment.

**Methods:**

We treated the cancer cells with MnO and Fe_2_O_3_ NPs and then exposed them to IR radiation for 6, 12, 24, 48, and 72 hours to investigate the effectiveness of this cancer treatment approach. To evaluate cytotoxicity, we conducted assessments on Skbr3 and HT29 cancer cells, both individually and in combination, using various methods.

**Results:**

The findings indicate that despite the inherent toxicity of NPs and IR laser radiation on cancer cells, the utilization of MnO and Fe_2_O_3_ NPs in conjunction with IR laser radiation treatment had the highest cytotoxic impact on cancer cells.

**Conclusions:**

These findings suggest that using MnO and Fe_2_O_3_ NPs in combination with IR laser therapy has great potential as an effective method for reducing the population of cancer cells.

This revision maintains the original content while ensuring clarity and adherence to the AMA style guidelines.

## 1. Background

Cancer is a multifaceted and devastating illness that disrupts the fragile equilibrium of the human body. With its diverse types and stages, cancer presents significant challenges, driving continuous research and innovation in prevention, diagnosis, and treatment to enhance outcomes and inspire hope for those impacted (1). Breast cancer is the most prevalent form of cancer among women worldwide, impacting millions of lives annually (2). Advances in early detection and treatment options have greatly increased survival rates for breast cancer, highlighting the crucial role of regular screenings and awareness initiatives (3, 4). Colorectal cancer is another frequently occurring type that affects the colon or rectum, components of the digestive system (5). It typically begins as small, noncancerous growths called polyps, which can develop into cancer over time (6). Risk factors for colorectal cancer include age, family history, certain genetic conditions, unhealthy lifestyle habits, and inflammatory bowel disease (7). Early detection through regular screenings such as colonoscopies can significantly improve treatment outcomes, as colorectal cancer is often highly treatable when detected at an early stage (8).

The treatment of cancer has made significant advancements in recent years, offering a range of options to target and combat the disease. Traditional treatments such as surgery, chemotherapy, and radiation therapy continue to play crucial roles in cancer management (9). New approaches like immunotherapy, targeted therapies, and precision medicine have emerged, providing personalized treatment strategies based on an individual's specific tumor characteristics (10, 11). Despite the progress made, there are still limitations in existing treatment options, including possible side effects, therapy resistance, a lack of comprehensive understanding of specific cancers, expensive treatment options, and restricted access to advanced therapies in certain areas (12). Continuous research and innovation are crucial to overcome these challenges and enhance cancer treatment results for patients globally.

Recently, the use of infrared (IR) laser technology in cancer treatment has emerged as a promising method (13). The IR lasers are capable of delivering precise and targeted energy to cancerous cells, resulting in localized hyperthermia and destruction of tumor tissue (14). The unique properties of IR lasers allow for deep tissue penetration while minimizing damage to surrounding healthy cells (15). This innovative approach holds great potential to improve cancer treatment outcomes by providing a minimally invasive, highly targeted, and efficient therapeutic option for patients (16). Combining IR laser therapy with other treatment modalities, such as the integration of IR laser therapy with the utilization of nanoparticles (NPs), can synergistically enhance their effectiveness (17). The NPs can selectively accumulate in tumor sites (18), acting as carriers for sensitizing agents or thermal enhancers (19). These NPs efficiently absorb and convert IR laser energy into heat, intensifying the destructive effects on cancer cells (20). Additionally, they can be engineered to carry anticancer drugs, enabling targeted delivery and enhancing the overall therapeutic outcome (21). The combined application of IR laser therapy and NPs holds immense potential in advancing the field of cancer treatment, offering improved efficacy, reduced side effects, and personalized therapeutic approaches for patients (22).

Manganese oxide (MnO) NPs, with their unique properties and versatility, have emerged as promising tools in medical applications. These NPs possess a high surface area-to-volume ratio, allowing for efficient drug loading and delivery systems. Their magnetic properties enable them to be used as contrast agents in imaging techniques such as magnetic resonance imaging (MRI). Moreover, manganese oxide NPs show potential in targeted therapy by selectively delivering therapeutic agents to specific cells or tissues, minimizing side effects. With ongoing research, these NPs hold great promise for revolutionizing medical diagnostics and treatment strategies (23-25).

Iron oxide (Fe_2_O_3_) NPs have gained significant attention in the field of medicine due to their unique properties. They possess magnetic properties, making them ideal for targeted drug delivery and imaging techniques like MRI. Their small size enables easy penetration through biological barriers, allowing for enhanced therapeutic efficacy. Additionally, Fe_2_O_3_ NPs have shown promise in hyperthermia treatment, where they generate heat when exposed to an external magnetic field, selectively killing cancer cells. Fe2O3 NPs hold tremendous potential for revolutionizing medical treatments and diagnostics (26-29).

The MnO and Fe_2_O_3_ have shown promising potential in the field of IR laser cancer treatment. These NPs possess unique physicochemical properties that make them suitable for targeted therapeutic interventions. When coupled with IR laser irradiation, these NPs can efficiently convert light energy into heat, resulting in localized thermal ablation of cancerous cells. The ability to precisely control the laser parameters allows for selective targeting of tumors while minimizing damage to healthy surrounding tissues. Furthermore, the biocompatibility and biodegradability of MnO and Fe_2_O_3_ NPs enhance their safety profile for clinical applications. With further research and development, the utilization of MnO and Fe_2_O_3_ NPs in IR laser cancer treatment holds great promise for enhancing the efficacy and precision of cancer therapies (30-32).

## 2. Objectives

In this study, we investigated the cytotoxic effects of IR laser therapy in conjunction with MnO and Fe_2_O_3_ nanoparticles on breast and colorectal cancer cell lines. To explore this proposed cancer treatment approach, the cancer cells were treated with MnO and Fe_2_O_3_ nanoparticles and subsequently exposed to IR radiation. Ultimately, we assessed the cytotoxicity of MnO, Fe_2_O_3_, and IR radiation both separately and in combination on breast and colorectal cancer cells through cytotoxicity evaluations.

## 3. Materials and Method

### 3.1. Materials

In this study, we utilized Fe_2_O_3_ and MnO NPs with an approximate size of 50 nm in diameter and concentrations of approximately 5.2 mg/mL and 6.6 mg/mL, respectively (purchased from Sigma Aldrich). We employed a 1064 nm IR laser (SmartFile, DEKA, Calenzano, Italy), the HT29 cell line (human colorectal adenocarcinoma cell line), and the Skbr3 cell line (breast cancer cell line). Additional materials included MTT (3-(4,5-dimethylthiazol-2-yl)-2,5-diphenyltetrazolium bromide), 10% fetal bovine serum (FBS), DMEM/F12 medium (Dulbecco's Modified Eagle Medium and Ham's Nutrient Mixture F-12), acridine orange solution, and penicillin/streptomycin (100 units/mL of penicillin and 100 μg/mL of streptomycin). Transmission electron microscopy (TEM) was also utilized.

This revision maintains the original content while ensuring clarity and adherence to the AMA style guidelines.

### 3.2. Methods

#### 3.2.1. Cell Culture

HT29 cell lines were cultured in DMEM/F12 medium (Gibco, USA) supplemented with 10% fetal bovine serum (FBS) and penicillin/streptomycin (100 units/mL of penicillin and 100 μg/mL of streptomycin). The cells were incubated at 37°C with 5% CO_2_ and used for experiments after 2 - 6 passages. Similarly, Skbr3 cell lines were cultured in DMEM/high glucose medium (Gibco, USA), also supplemented with 10% FBS and penicillin/streptomycin (100 units/mL of penicillin and 100 μg/mL of streptomycin). These cells were maintained under the same incubation conditions (37°C with 5% CO₂) and used after 2 - 6 passages. The cell culture protocol for the Skbr3 cell line followed the same procedure as that of the HT29 cell line (33). To evaluate cytotoxicity, six groups of cells were prepared by treating them with MnO, Fe_2_O_3_, and IR laser radiation, including Tc (control, without any treatment), TF (treated with Fe_2_O_3_), TMn (treated with MnO), TL (treated with IR laser radiation), TL.Mn (treated with IR laser radiation in combination with MnO), and TL.Fe (treated with IR laser radiation in combination with Fe_2_O_3_).

#### 3.2.2. Infrared Laser Radiation to Cancer Cells

A semiconductor laser (Yenista Optics, OSICS T100 Tunable Laser Module T100 1310) with a tuning range of 1260 - 1360 nm was used as the irradiation source. The average output power was 4 mW, with a linewidth of less than 1 nm and a wavelength stability of 0.1 nm/h. The irradiation was delivered using a fiber patch cord equipped with an air-spaced doublet collimator at the end. The fiber collimator featured a non-magnetic stainless steel housing and was pre-aligned to collimate the laser beam emitted from the tip of an FC/PC connectorized fiber, ensuring diffraction-limited performance at the design wavelength. The surface dose (energy density) of laser radiation absorbed by biological tissue (E, J/cm^2^) was calculated using the formula: E = Pt/S, where P is the average output power (W), t is the exposure time (seconds), and S is the laser spot area on the cell culture (cm^2^). For IR irradiation of cancer cells, 5 × 10^4^ HT29 and Skbr3 cells across six groups (Tc, TIR, TS, TM, TIR-S, and TIR-M) were seeded onto a 12-well plate and incubated at 37°C in a humidified atmosphere containing 5% CO_2_. The laser source was positioned beneath the plate at a distance of 0.5 cm from the slide chamber. While one group of cells was irradiated, the control group was shielded using a steel foil. Laser energy densities ranging from 0.3 to 9.45 J/cm² were applied depending on the exposure time (34).

#### 3.2.3. Cell Viability

To assess the viability of cancer cells, 5 × 10^4^ HT29 and Skbr3 cells were seeded into six groups (T_c_, T_F_, T_Mn_, T_L_, T_L.Fe_, and T_L.Mn_) on a 12-well plate. The cells were incubated at 37°C in a humidified atmosphere containing 5% CO₂. Following treatment, cell viability was evaluated at 6, 12, 24, 48, and 72 hours. At each time point, the culture medium was removed, and 100 μL of MTT solution (0.5 mg/mL) was added to each well. The plate was then incubated for 4 hours. After incubation, the MTT solution was replaced with 100 μL of DMSO and incubated for an additional 10 minutes to dissolve the formazan crystals. The absorbance was subsequently measured at 570 nm using an ELISA reader (Infinite 200 M, Tecan, Basel, Switzerland) (35).

#### 3.2.4. Lysosomal Membrane Integrity Assay

To evaluate lysosomal membrane integrity, we employed the acridine orange redistribution assay. Acridine orange is a fluorescent dye that selectively accumulates in acidic cellular compartments, particularly lysosomes. In intact lysosomes, the dye concentrates and emits green fluorescence. However, when lysosomal membranes are compromised, acridine orange leaks into the cytoplasm and binds to other acidic organelles, such as mitochondria, resulting in redistribution of the dye and an increase in red fluorescence intensity (36).

To assess this, cells were treated at various time points (6, 12, 24, 48, and 72 hours). Following treatment, a suspension of pre-stained cells from each group (T_c_, T_F_, T_Mn_, T_L_, T_L.Fe_, and T_L.Mn_) was prepared in the presence of acridine orange (5 mM). The cell suspension was collected by retrieving the dye-containing incubation medium and centrifuging at 800 g for 1 minute. The resulting cell pellet was resuspended in 2 mL of fresh incubation buffer to ensure accurate fluorescence measurements. To eliminate any residual extracellular dye, the cells were washed twice.

Fluorescence measurements were performed using a fluorimeter (Shimadzu RF-5000, Japan), with the excitation wavelength set at 495 nm and the emission wavelength at 530 nm, enabling assessment of dye redistribution and lysosomal membrane integrity (37).

#### 3.2.5. Lipid Peroxidation Assay

The amount of thiobarbituric acid reactive substances (TBARS) formed was used to assess the lipid peroxidation (LPO) content in all experimental groups. For this purpose, 1 × 10^6^ cells/mL from each group were used. The LPO content was measured using an ELISA reader (Infinite 200 M, Tecan, Basel, Switzerland) at an absorbance of 532 nm. Each sample was tested in triplicate to ensure reproducibility and accuracy of the results (35).

#### 3.2.6. Protein Carbonyl Assay

First, proteins were precipitated by adding an equal volume of 20% trichloroacetic acid (TCA), followed by centrifugation at 11,000 × g for 5 minutes. The resulting cell pellet (1 × 10^6^ cells/well) was then resuspended in 2,4-dinitrophenylhydrazine (DNPH) solution (10 mmol/L) and incubated at room temperature for 15 - 30 minutes. Subsequently, 20% TCA was added again, and the samples were centrifuged at 11,000 × g for 3 minutes. The protein carbonyl content was measured using an ELISA reader (Infinite 200 M, Tecan, Basel, Switzerland) at an absorbance of 450 nm (35).

### 3.3. Statistical Analysis

All statistical analyses were performed in triplicate using Prism version 5 software. Data were expressed as mean ± standard deviation (SD). Statistical significance was defined as P < 0.05. One-way ANOVA followed by Tukey’s post hoc test and two-way ANOVA followed by Bonferroni’s post hoc test were used to assess differences between groups.

## 4. Results

### 4.1. Characterization of Manganese Oxide and Iron (III) Oxide

**Figure 1.**
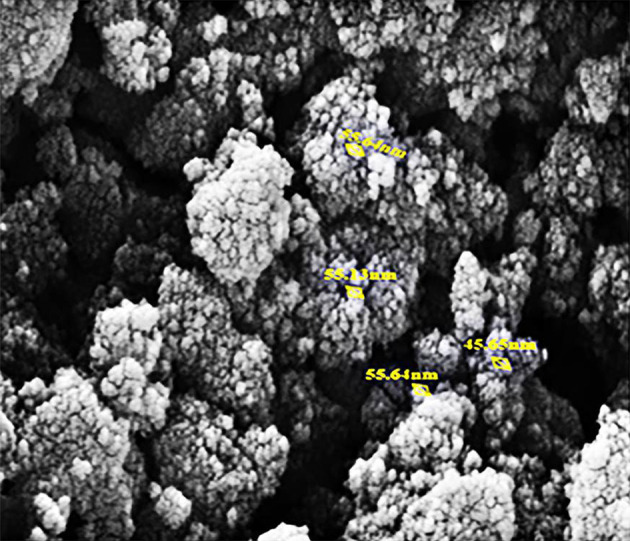
SEM micrograph of manganese oxide (MnO) nanoparticles (NPs)

**Figure 2.**
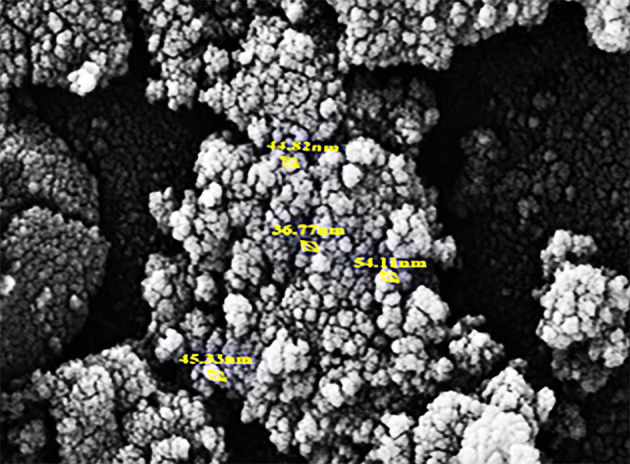
SEM micrograph of iron (III) oxide (Fe_2_O_3_) nanoparticles (NPs)

For the characterization of NPs, we utilized SEM analysis. Figures 1 and 2 is related to the SEM micrograph of MnO and Fe_2_O_3_ NPs respectively.

### 4.2. Cell Viability

**Figure 3.**
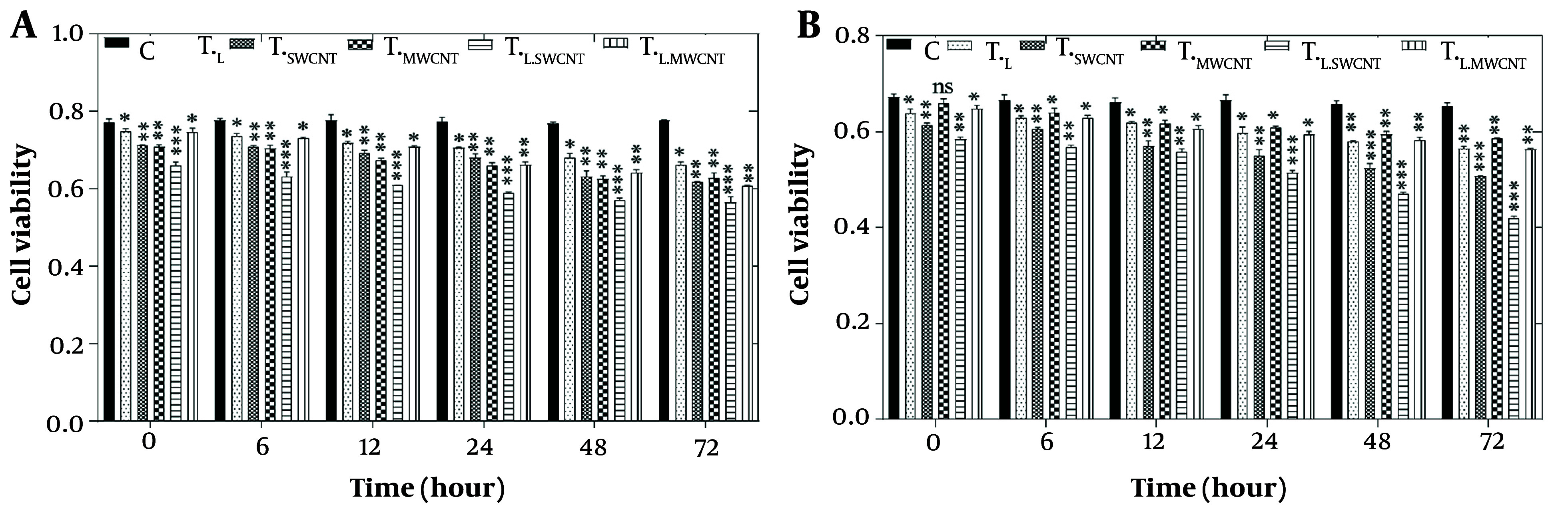
MTT assay results of HT29 (A) and Skbr3 cancer (B), cancer cells in 6 groups including control, T_F_, T_Mn_, T_L_, T_L.Fe_, and T_L.Mn_. The cells were treated with infrared (IR) radiation and nanoparticles (NPs) for different durations of 6, 12, 24, 48, and 72 h. Data shown as mean ± SD. *, ** and *** represent P < 0.05, P < 0.01 and P < 0.001 (respectively) vs. control group. Ns = no significant.

To evaluate the effects of laser treatment and MnO and Fe₂O₃ nanoparticles on HT29 and Skbr3 cancer cells, the MTT assay was applied to six experimental groups: Control (T_c_), T_F_, T_Mn_, T_L_, T_L.Fe_, and T_L.Mn_. The results of the MTT assay for HT29 cells across these six groups at 6, 12, 24, 48, and 72 hours post-treatment are presented in Figure 3A. A significant reduction in cell viability (P < 0.05) was observed in all treatment groups at 6, 12, 48, and 72 hours compared to the control group.

Similarly, the MTT assay results for Skbr3 cells at the same time points are shown in Figure 3B. Cell viability in all treatment groups was significantly reduced at 6, 12, 24, 48, and 72 hours when compared to the control group (P < 0.05).

### 4.3. Lysosomal Membrane Integrity Assay

**Figure 4.**
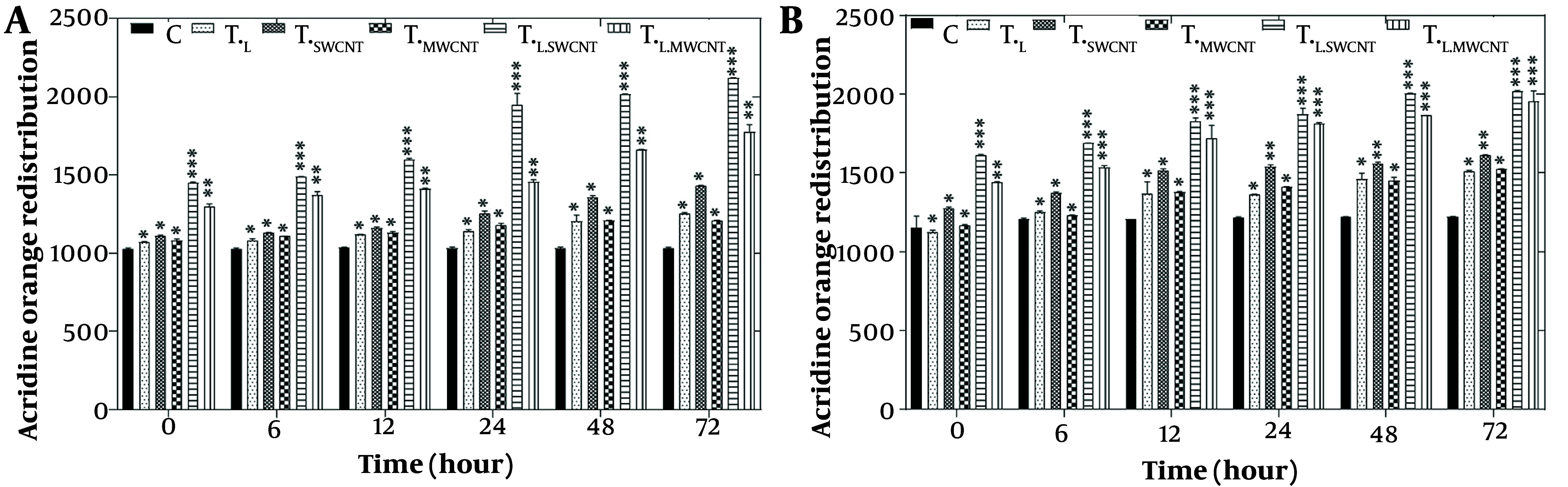
Lysosomal membrane integrity evaluation via acridine orange redistribution results of HT29 (A) and Skbr3 cancer (B), cancer cells in 6 groups including control, T_F_, T_Mn_, T_L_, T_L.Fe_, and T_L.Mn_. The cells were treated with infrared (IR) radiation and nanoparticles (NPs) for different durations of 6, 12, 24, 48, and 72 h. Data shown as mean ± SD. *, ** and *** represent P < 0.05, P < 0.01 and P < 0.001 (respectively) vs. control group. Ns = no significant.

The evaluation of lysosomal membrane integrity was performed using the acridine orange redistribution assay. For this assay, cells were treated with laser radiation and MnO or Fe_2_O_3_ nanoparticles for 6, 12, 24, 48, and 72 hours across six groups: T_c_, T_F_, T_Mn_, T_L_, T_L.Fe_, and T_L.Mn_. The results showed a significant increase in acridine orange redistribution (P < 0.05), indicating lysosomal membrane permeabilization in both the HT29 cell line (Figure 4A) and Skbr3 cancer cells (Figure 4B) at all time points across the six treatment groups.

### 4.4. Lipid Peroxidation Assay

**Figure 5.**
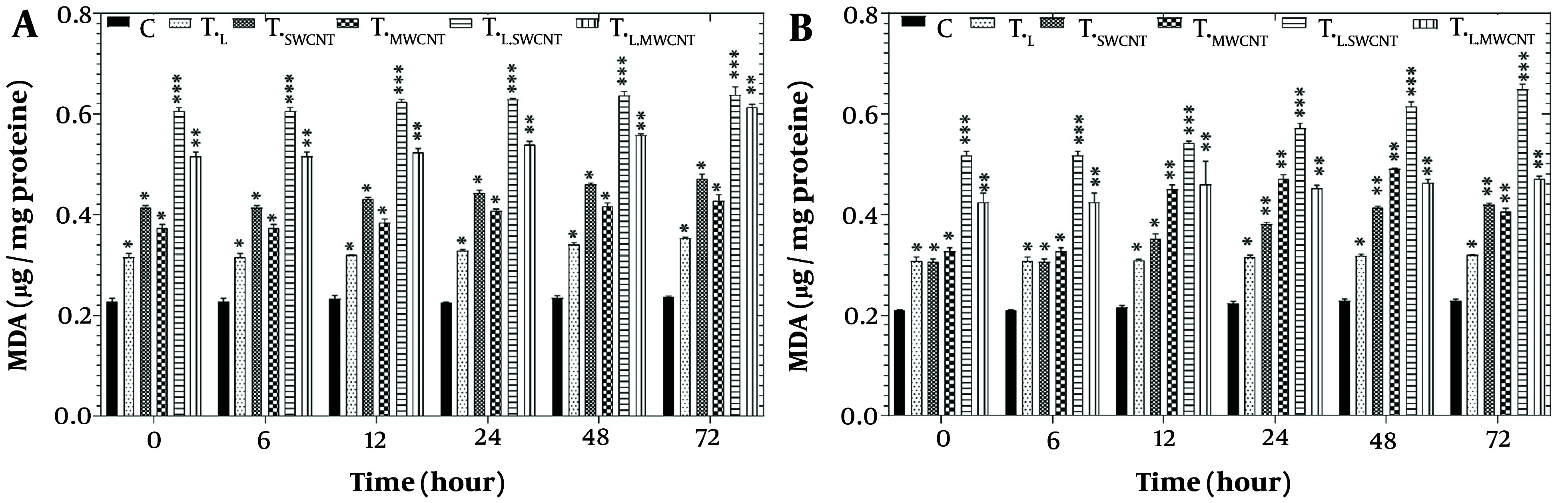
Lipid peroxidation assay by TBARS results of HT29 (A) and Skbr3 cancer (B), cancer cells in 6 groups including control, T_F_, T_Mn_, T_L_, T_L.Fe_, and T_L.Mn_. The cells were treated with infrared (IR) radiation and nanoparticles (NPs) for different durations of 6, 12, 24, 48, and 72 h. Data shown as mean ± SD. *, ** and *** represent P < 0.05, P < 0.01 and P < 0.001 (respectively) vs. control group. Ns = no significant.

Figure 5 presents the results of the LPO assay in HT29 (Figure 5A) and Skbr3 (Figure 5B) cell lines across six groups (T_c_, T_F_, T_Mn_, T_L_, T_L.Fe_, and T_L.Mn_) following treatment with laser radiation and MnO or Fe_2_O_3_ nanoparticles for 6, 12, 24, 48, and 72 hours. A significant induction of LPO (P < 0.05) was observed in both HT29 and Skbr3 cells in all treatment groups at each time point compared to the control.

### 4.5. Protein Carbonyl Assay

**Figure 6.**
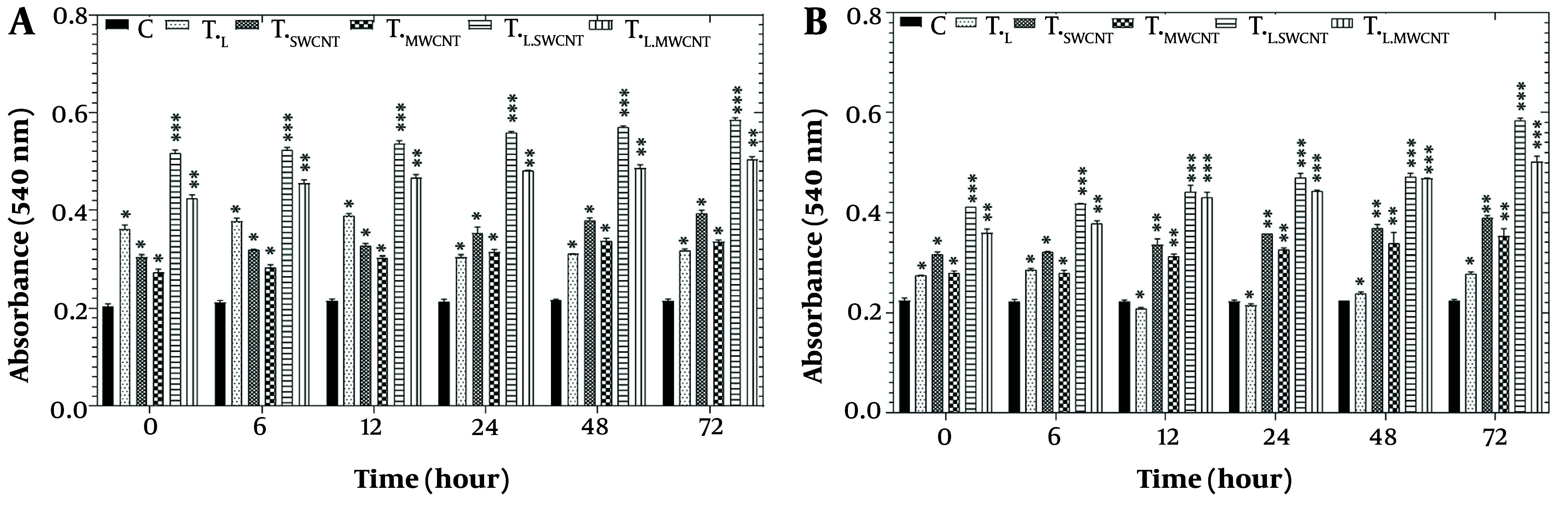
Protein carbonyl results of HT29 (A) and Skbr3 cancer (B) cancer cells in 6 groups including control, T_F_, T_Mn_, T_L_, T_L.Fe_, and T_L.Mn_. The cells were treated with infrared (IR) radiation and nanoparticles (NPs) for different durations of 6, 12, 24, 48, and 72 h. Data shown as mean ± SD. *, ** and *** represent P < 0.05, P < 0.01 and P < 0.001 (respectively) vs. control group. Ns = no significant.

Figure 6 shows the results of the protein carbonyl assay in HT29 (Figure 6A) and Skbr3 (Figure 6B) cell lines across six groups (T_c_, T_F_, T_Mn_, T_L_, T_L.Fe_, and T_L.Mn_) following treatment with laser radiation and MnO or Fe_2_O_3_ nanoparticles for 6, 12, 24, 48, and 72 hours. A significant increase in protein carbonyl content (P < 0.05) was observed in both HT29 and Skbr3 cells across all treatment groups and time points compared to the control group.

## 5. Discussion

In this study, we explored the use of MnO and Fe_2_O_3_ NPs as targeted therapeutic agents for the disruption of cancer cell function. Conventional cancer therapies are often limited by challenges such as drug resistance, off-target effects, and serious adverse events, highlighting the urgent need for strategies that selectively target cancer cells while sparing healthy tissues. Mitochondria, essential for cellular energy production, apoptosis regulation, and reactive oxygen species (ROS) generation, have emerged as key therapeutic targets in cancer treatment (35).

Our approach involved treating colorectal (HT29) and breast (Skbr3) cancer cells with MnO and Fe_2_O_3_ NPs, followed by exposure to IR laser radiation. Cell viability was assessed using the MTT assay in six experimental groups: T_c_, T_F_, T_Mn_, T_L_, T_L.Fe_, and T_L.Mn_. As shown in Figure 3A and B, cell viability decreased over time in both HT29 and Skbr3 cells, with the lowest viability observed at 72 hours. The combination of IR laser radiation and NPs produced the greatest cytotoxic effect in both cell lines. Among the individual treatments, Fe_2_O_3_ NPs showed greater cytotoxicity than MnO NPs or IR radiation alone. Notably, Skbr3 breast cancer cells exhibited higher sensitivity to treatment compared to HT29 colorectal cancer cells.

Supporting evidence comes from Razumov et al., who demonstrated selective cytotoxicity of MnO NPs against human glioblastoma cells while sparing healthy cells. Their study also identified the activation of cell death signaling pathways induced by the nanoparticles (38). Similarly, another study confirmed significant cytotoxicity of MnO NPs in HT29 cells through MTT assays (39). Alarifi et al. showed that Fe_2_O_3_ NPs induced cytotoxic and genotoxic effects in MCF-7 breast cancer cells via ROS production, lipid peroxidation, decreased antioxidant enzyme activity, nuclear fragmentation, apoptosis, and caspase-3 activation (40).

The IR laser radiation, known for its potential to induce DNA damage and trigger programmed cell death, was shown in a separate study to significantly increase apoptosis in pancreatic cancer cells. The addition of gemcitabine to near-infrared (NIR) laser therapy synergistically enhanced apoptotic effects (41).

Nanoparticles have been widely reported to generate ROS in cancer cells, elevating oxidative stress levels (23). Reactive oxygen species, including superoxide (O₂⁻) and hydrogen peroxide (H_2_O_2_), are byproducts of mitochondrial respiratory chain activity and can damage cellular macromolecules, particularly lipids, proteins, and DNA (42). This oxidative damage disrupts mitochondrial electron transport chains and triggers apoptosis or necrosis. A direct correlation between TBARS formation and LPO was observed in HT29 and Skbr3 cells following treatment with MnO, Fe_2_O_3_, and IR radiation, as shown in Figure 5A and B.

Protein carbonylation, another marker of oxidative damage, also increased in a time-dependent manner in both cell lines (Figure 6A and B) following treatment. This increase may be associated with the release of pro-apoptotic proteins such as cytochrome c, which play a critical role in cell death signaling pathways (42). Reactive oxygen species-induced protein oxidation alters amino acid side chains, resulting in carbonyl formation. Protein carbonylation has been implicated in endoplasmic reticulum and lysosomal stress, aging, and antioxidant depletion (42, 43).

Lysosomal membrane integrity was assessed using the acridine orange redistribution assay, as illustrated in Figure 4A and B. Cells were treated across the six groups (T_c_, T_F_, T_Mn_, T_L_, T_L.Fe_, and T_L.Mn_) for 6, 12, 24, 48, and 72 hours. Treatment with MnO, Fe_2_O_3_, and IR laser radiation led to redistribution of acridine orange from lysosomes to the cytoplasm, evidenced by a time-dependent increase in red fluorescence. The most significant redistribution occurred at 72 hours, indicating extensive lysosomal membrane permeabilization and cytotoxicity.

These findings confirm that the combined application of NPs and IR radiation induces greater cytotoxicity than any single treatment. This was consistent with MTT results. Previous studies have also employed acridine orange staining to evaluate lysosomal integrity. One such study using magnetic systems and iron oxide NPs on liver cancer cells found increased cathepsin B activity, supporting lysosomal membrane permeabilization and apoptosis induction (42). Ramu et al. developed a binuclear platinum (II) BODIPY complex for lysosomal targeting and near-IR-induced photocytotoxicity. Using acridine orange assays, they demonstrated lysosomal accumulation and potent photodynamic apoptotic activity in cancer cells, with minimal toxicity in the absence of light (43).

Previous studies indicate that NPs significantly impair mitochondrial function by modulating ROS dynamics, leading to disrupted ATP production and elevated levels of ROS (44). This mechanism corresponds with observations suggesting that NPs utilize oxidative stress and osmotic pressure to trigger pyroptosis in tumor cells. Targeted inhibition of mitochondrial complexes can push ROS levels beyond cellular repair thresholds, thereby threatening tumor cell viability.

Our research supports this mechanism, showing that NPs designed to target specific mitochondrial complexes not only elevate ROS levels but also enhance immune responses by promoting immunogenic cell death (ICD) and facilitating T-cell infiltration into hypoxic tumor regions (44). These findings align with recent studies demonstrating that programmed drug release in such microenvironments can amplify immune system activation. Notably, the inhibition of mitochondrial oxidative stress has been shown to generate ROS levels consistent with the "ROS storm" model, inducing oxidative damage and potentially activating ICD pathways, which initiate immune responses against residual tumor cells (44).

Moreover, our results suggest that ROS-based therapies may overcome the barriers posed by hypoxic and acidic tumor microenvironments, which often limit ROS generation in conventional therapies. By targeting both mitochondrial and cytoplasmic ROS production, this approach may yield more consistent therapeutic outcomes. The persistent oxidative imbalance observed could serve as a preparatory phase for chemoimmunotherapy. For instance, the Na_2_S_2_O_8_ nanoparticle system has been shown to disrupt cellular osmolarity via Na⁺ ion release, thereby disturbing ion homeostasis and sensitizing cancer cells to therapy.

Additionally, tailoring the physicochemical properties of NPs to activate diverse cell death pathways — such as pyroptosis — presents an exciting opportunity to exploit inflammatory signaling and further enhance anti-tumor immunity. In particular, our findings demonstrate that NPs selectively disrupt oxidative stress regulation, mirroring mechanisms involved in the metabolic collapse of therapy-resistant cancer cells via inhibition of mitochondrial oxidative phosphorylation (OXPHOS). This strategy holds significant promise, especially given the reprogrammed metabolic nature of cancer cell mitochondria, which support rapid proliferation and resistance development (44).

While this study offers valuable insights into the therapeutic potential of NPs for inducing cell death in cancer cells, several limitations must be acknowledged. First, the study is based primarily on in vitro data derived from cell lines. In vivo validation is necessary to confirm the efficacy and safety of these nanoparticle treatments. Additionally, although ROS induction was observed, the relatively small dataset limited detailed comparisons across different types of nanoparticles (45). The study also focused on mitochondrial and lysosomal effects, without fully addressing interactions with other organelles or broader cellular pathways that could result in off-target effects.

Long-term consequences of NP exposure were not investigated, particularly regarding their accumulation and clearance within the body — key factors in determining biocompatibility. Furthermore, the mechanisms of NP uptake and mitochondrial targeting across different cancer types were not examined, limiting the generalizability of our findings.

Future research should aim to address these limitations. In vivo studies are essential for evaluating nanoparticle pharmacokinetics, biodistribution, clearance, and long-term safety profiles. Investigations into the synergistic use of NPs with conventional chemotherapy or immunotherapy — especially in drug-resistant cancers — may uncover new therapeutic avenues. Developing environmentally responsive nanocarriers, such as those sensitive to pH or redox conditions, could enhance specificity for the acidic, hypoxic tumor microenvironment, thereby improving treatment accuracy.

One promising direction is to explore NP – cancer cell interactions more deeply, particularly for preventing tumor recurrence and metastasis. For example, mitochondrial inhibitors like Gboxin — which preferentially target OXPHOS in glioblastoma cells—demonstrate that mitochondrial disruption can achieve significant anti-tumor effects while sparing healthy cells (46, 47). These findings collectively highlight the vast potential of NPs in advancing cancer therapeutics through precise mitochondrial targeting and oxidative stress modulation.

### 5.1. Conclusions

In this study, we investigated the cytotoxic effects of combining IR laser therapy with MnO and Fe_2_O_3_ NPs on cancer cells. Our findings suggest that this combination induces significant cytotoxicity, supporting its potential as a targeted therapeutic strategy. To further enhance efficacy and minimize off-target effects, future research should focus on developing NPs with functional groups designed to specifically target oxidative stress pathways reprogrammed by oncogenes.

Additionally, a more comprehensive examination of the effects of NPs on other cellular organelles and metabolic pathways is essential for understanding the broader systemic impact of nanoparticle-based cancer therapies. Despite the promising in vitro results, further in vivo studies are necessary to validate the effectiveness, safety, and translational potential of this strategy in clinical cancer treatment.

## Data Availability

The data presented in this study are uploaded during submission as a supplementary file and are openly available for readers upon request.
